# Contribution of community health workers to improving access to timely and appropriate case management of childhood fever in Mozambique

**DOI:** 10.7189/jogh.07.010402

**Published:** 2017-06

**Authors:** Tanya Guenther, Salim Sadruddin, Karen Finnegan, Erica Wetzler, Fatima Ibo, Paulo Rapaz, Jeanne Koepsell, Ibad ul Haque Khan, Agbessi Amouzou

**Affiliations:** 1 Save the Children US, Washington, DC, USA; 2 World Health Organization, Geneva, Switzerland; 3 Johns Hopkins University, Institute of International Programs, Baltimore, Maryland, USA; 4 Save the Children International, Maputo, Mozambique; 5 Save the Children International, Islamabad, Pakistan

## Abstract

**Background:**

Large scale evaluations in several settings have demonstrated that lay community health workers can be trained to provide quality case management of childhood illnesses. In 2010, Mozambique introduced the integrated community case management (iCCM) strategy to reach children in remote areas with care provided through *Agentes Polivalentes Elementares* (APEs). We assessed the contribution of the program to improved care–seeking and appropriate treatment of childhood febrile illness in Nampula Province.

**Methods:**

We used a post–test quasi–experimental design with three intervention and one comparison districts to compare access and appropriateness of care for sick children in Nampula province. We carried out a household survey in the study districts to measure levels of care–seeking and treatment of childhood fever after approximately two years of full implementation of the iCCM program in the intervention districts. We also assessed consistency of care with standard case management protocols comparing children receiving care from (APEs) to those receiving care from first–level health facilities.

**Results:**

A total of 773 children 6–59 months with fever in the last two weeks were included in the study. In iCCM served areas, APEs were the predominant source of care and treatment; 87.1% (95% confidence interval CI 80.8–93.4) of children 6–59 months with fever who sought care were taken first to an APE and APEs accounted for 86.2% (95% CI 79.7–92.7) of all first–line antimalarial treatments. Public health facilities were the leading source of care in comparison areas, providing care to 86.1% (95% CI 79.0–93.3) of children with fever taken for care outside the home. Timeliness of treatment was significantly better in intervention areas, where 63.9% (95% CI 54.4–73.3) of children received treatment within 24 hours of symptom onset compared to 37.5% (95% CI 31.1–43.9) in comparison areas. Children taken first to an APE were more likely to receive a rapid diagnostic test (RDT) (68.1%; 95% CI 57.2–79.0) and to have their respiratory rate assessed (60.0%; 95% CI 45.4–74.6) compared to children taken to health facilities (41.4%; 95% CI33.7–49.2 and 19.4%; 95% CI 8.4–30.5, respectively). Overall, 61.3% (95% CI 51.5–71.0) of children with fever receiving care from APEs received the correct drug within 24 hours and for the correct duration compared to 26.0% (95% CI 18.2–33.9) of those receiving care from health facilities.

**Conclusion:**

iCCM contributed to improved timely and appropriate treatment for fever for children living far from facilities. Trained, supplied and supervised APEs provided care consistent with iCCM protocols and performed significantly better than first level facilities on most measures of adherence to case management protocols. These findings reinforce the need for comprehensive efforts to strengthen the health system in Mozambique to enable reliable support for quality of case management of childhood illness at both health facility and community levels.

An estimated 5.9 million children die each year before reaching their fifth birthday; about half of these deaths are caused by infectious diseases [1]. Among children aged 1 to 59 months, pneumonia, diarrhea, and malaria remain leading causes of death, responsible for 1.8 million child deaths annually [2]. Integrated community case management (iCCM) seeks to reduce the mortality burden among children under five by improving access to equitable, life–saving interventions that address the leading causes of death such as pneumonia, malaria and diarrhea [3]. It relies on short–term training (generally one week), equipping and supervision of lay community workers to provide care for uncomplicated cases of pneumonia, malaria and diarrhea and referral for complicated cases among children age 2–59 months. In 2012, the World Health Organization (WHO) and United Nations Children’s Fund issued a joint statement on the role of iCCM in reducing under–five mortality through community–based care for malaria, diarrhea, and pneumonia [3]. The joint statement urged governments adopting iCCM to support programs with adequate training, a strong supply chain, and ongoing monitoring of activities, all built upon existing infrastructure and systems [3]. As of 2014, iCCM for malaria, diarrhea and pneumonia was being implemented in 28 countries in sub–Saharan Africa, including Mozambique [4].

Mozambique is one of just 12 low–income countries to achieve its Millennium Development Goal (MDG) of reducing child mortality by two–thirds or more between 1990 and 2015 [1]. In 1990, Mozambique’s under–five mortality rate was estimated to be 240 per 1000 live–births and by 2015 this had dropped to an estimated 79 per 1000 live–births [1]. Malaria is the leading cause of death among children aged 1–59 months followed by pneumonia, HIV, and diarrhea [5]. Mozambique formally introduced iCCM into the national health system in 2010, through a national program of community health workers (CHWs) referred to as *Agentes Polivalentes Elementares* (APEs) who provide iCCM services to communities located 8–25km from the nearest health facility [6,7].

Although there is global evidence that CHWs are capable of providing quality iCCM services [8–10], there are some concerns within Mozambique regarding the ability of APEs to provide quality care and the extent to which iCCM will contribute to improved treatment coverage [11,12]. Despite endorsing iCCM nationally, the Ministry of Health (MOH) in Mozambique emphasizes the health promotion activities of the APEs, recommending that APEs spend 80% of their time conducting home visits and community meetings and just 20% of their time for curative activities including iCCM [12]. While large scale evaluations from other settings have shown that CHWs implementing iCCM within existing government systems can provide quality care and contribute to increases in treatment coverage [13–15], there is limited evidence from the iCCM program in Mozambique regarding the performance of APEs implementing iCCM that can help inform national implementation and financing decisions [12].

Save the Children (SC), with funding from the Canadian International Development Agency (now called Global Affairs Canada) provided support to the Mozambican MOH for implementing iCCM from 2009 to 2013, with a focus on malaria (fever) management, the leading cause of child deaths. We present the findings of a quasi–experimental evaluation of the APE iCCM program that compares levels of care–seeking and timely and appropriate treatment coverage for fever in an intervention area with a well–supported iCCM program to levels in a comparison area where the APE iCCM program had yet to be implemented. We also compare consistency of care provided by APEs and first level health facilities with established cases management protocols.

## Program description

## Mozambique’s revitalized APE program

In Mozambique, community–based health services are provided through the APEs. The APE cadre was established in 1978, but services were disrupted due to the protracted civil war, which ended in 1992 [6]. In 2010, the MOH set out to revitalize its community health worker program, aiming to train and deploy 5000 APEs across Mozambique’s ten provinces – with an initial target of 25 APEs per district [6,7]. Prior to 2010, APEs were implementing community case management for some illnesses including malaria and diarrhea, but support for implementation was confined to small areas with partner support and there was no standardized training package endorsed under national MOH policies. Under the revitalized program, APEs located 8 to 25 km from the nearest health facility are designated for training in the revised national package and are meant to serve a target catchment population of 500–2000 individuals [6,7]. The national APE program is managed by the Department of Health Promotion, which coordinates technical oversight, program financing, monitoring and evaluation, and liaises with other departments in the MOH, donors and implementing partners [6]. While officially considered volunteers, the APEs receive a monthly stipend of 1200 meticais (equivalent to approximately US$ 40 at the time of the study) set by the Ministry of Health, with implementing partners being responsible to cover the stipend in the areas they support iCCM implementation [6].

Under the revitalized program, APEs receive 18 weeks of training organized into four blocks; iCCM is covered for one week during the third block [6]. APEs are trained in the assessment, classification, and treatment of common childhood illnesses, including fever, diarrhea, and suspected pneumonia, based on WHO’s standard protocol for management of childhood illness by CHWs and MOH protocols. In addition to providing case management to children under–five, APEs treat other non–ICCM conditions including conjunctivitis and scabies, identify and refer children with moderate to severe acute malnutrition, and conduct home visits and community health promotion talks to relay health promotion and disease prevention messages. The APEs also deliver first aid and provide treatment for malaria and diarrhea to community members older than five years of age.

## Case management of childhood illness in Mozambique

Under the iCCM protocol, APEs assess children 2–59 months for general danger signs as a first step [16]. If general danger signs are present, APEs refer patients to government–run first level health facilities referred to as *Unidade Sanitária*. Children presenting with fever or a history of fever are tested for malaria with a rapid diagnostic test. Children between 6–59 months with a positive RDT for malaria receive artemether–lumefantrine (AL) and paracetamol from the APE. A child with a negative test is given paracetamol, and referred to the first level health facility. Children 2–59 months with cough/difficult breathing are assessed for fast breathing by counting breathes for one minute using a digital timer. A child with respiratory rate above the WHO age–specific cut–off point is classified as fast–breathing pneumonia and treated with amoxicillin dispersible tablets. Children aged 2–59 months presenting with diarrhea are treated with recommended doses of ORS and zinc.

At first level health facilities, providers follow a similar algorithm based on WHO’s Integrated Management of Childhood Illness (IMCI) protocol. According to the protocol, all children presenting with fever should be tested for malaria with an RDT and if positive, given the first–line antimalarial (AL). Fever is treated with either paracetamol in suspension or tablets. Those presenting with cough/difficult breathing should be assessed by counting breaths for one minute using a timer (or if not available a watch or mobile phone). Under MOH protocols, those with fast–breathing pneumonia receive amoxicillin or cotrimoxazole in suspension (MOH protocol was amended in early 2011 to include amoxicillin as first line treatment). Children with diarrhea should receive ORS and zinc. First level health facilities typically provide IMCI services 5 days per week (Monday to Friday) from 7:00am to 3:00pm. However, health staff live on the facility grounds so in theory, they can attend to severely ill children or those with danger signs at any time. The first level facilities are usually staffed with a medical technician (Técnico de Medicina), or/and a general nurse or MCH nurse. Both nurses and medical technicians receive 30 months training. Medicines and supplies for treatment of child illness (RDTs, AL, amoxicillin or cotrimoxazole syrup, paracetamol pills or syrup, ORS and zinc) are meant to be provided to facilities monthly through provincial and district medical stores.

## The iCCM program supported by Save the Children

Save the Children provided support for iCCM implementation, with a focus on fever management, in 10 districts in Nampula province and five districts in Gaza province from 2009 to 2013. APEs satisfied national eligibility criteria including the ability to read and write in Portuguese and complete basic arithmetic. In 2010, SC trained and equipped 319 APEs across the 15 districts to deliver iCCM services. These APEs were trained before the MOH had finalized the curriculum and manuals for the revitalized APE program, a task which was completed in October 2011. Thus, the first group of APEs trained by SC received six days of iCCM training based on training modules developed by SC together with the MOH. The training included a mix of classroom exercises and clinical practical sessions to provide the necessary knowledge and skills related to iCCM. Later, this iCCM module was adapted and incorporated into the larger, national APE curriculum and training package. The APEs received a week long refresher training in March 2012.

Training activities were implemented using a cascade model, in which SC and MOH master trainers trained health facility staff and SC–employed district coordinators, who in turn trained the APEs At the end of the training, APEs were administered a clinical competency assessment and APEs who passed were awarded certificates upon completion of the training and provided a kit of essential drugs and supplies. The APEs kept the kit at the health post, which served as iCCM delivery site as well. The kit included: medicines specified by the national iCCM protocol including AL, amoxicillin, ORS, and zinc; respiratory timers; job aids; and a treatment register. The APEs received stipend support from Save the Children and MOH (via external funding from other donors).

During the project implementation period, SC provided technical support including development and adaptation of tools, training, monitoring, and supervision. Save the Children trained MOH staff at district and health facility levels in supervision and supply chain support. APEs were supervised monthly within their communities by district and facility based MOH staff trained on iCCM protocols, monitoring and supervision. SC placed staff at district level to support and mentor the MOH staff to supervise and monitor APEs. AL, amoxicillin, and malaria RDTs were procured by SC through local suppliers approved by the MoH. The medicines procured by SC were supplemented with medicines (ORS, AL) from the MOH system. Additionally, SC transported these medicines and supplies from the provincial capital to the districts and, in some cases, to the APEs’ reference health facility. Medicines were procured and distributed to the APEs based on number of cases treated reported by APEs.

## METHODS

### Study setting

**Figure 1:**
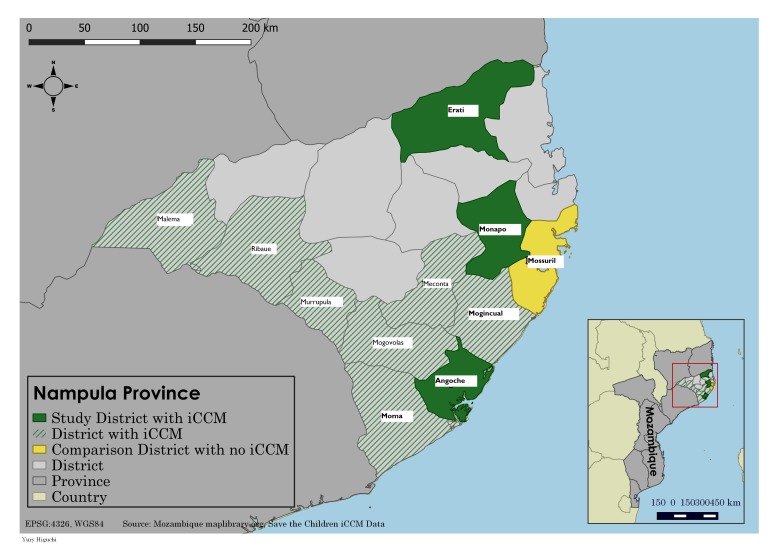
iCCM (integrated community case management) implementation supported by Save the Children and evaluation areas, Nampula province, Mozambique.

Nampula province is located in the north of Mozambique and, as of 2013, had an estimated population of more than 4.7 million. Nampula province was chosen for the endline study given that more than 90% of under–five children targeted through SC support were located within the province. We conducted a household survey in areas with iCCM services in three intervention districts (Angoche, Erati and Monapo) that had received SC support and one comparison district, Mossuril, where the APE revitalization and iCCM program had yet to be rolled out (**Figure 1**).

Angoche, Erati and Monapo were selected for the evaluation as they were the first districts in Nampula to receive iCCM training and had initiated full implementation by late 2010. The three districts had a total population of 837 245 and were served by 41 government health facilities. The districts had a high number of iCCM–trained APEs; a total of 157 APEs were trained and deployed in iCCM across these three districts, serving an estimated population of 329 752 (an average of 2213 per APE) and 61 158 children under five. Mossuril, the comparison district, was selected for the evaluation as it was geographically proximate to the intervention areas (located adjacent to Monapo district) and had a similar health service profile, with a total population of 119 223 with 10 government health facilities.

### Study design and sampling

A cross–sectional household survey was conducted in Nampula province in November 2012 after approximately two years of program implementation. The sample size of 600 households from the three intervention districts and 600 households from the comparison district was powered to detect a 20% difference in fever (presumptive malaria) treatment between intervention and comparison areas, assuming a fever prevalence of 27% and baseline treatment of 42%. Households with children aged 2–59 months were selected using a two–stage sampling procedure. In intervention districts, 30 APE catchment areas were selected with equal probability (ten per district) and in the comparison areas 30 census enumeration areas that were at least 8 km from nearest health facility (and therefore eligible for implementation of iCCM program) were sampled proportional–to–size from a listing of all eligible enumeration areas. A mapping team sketched basic maps of each selected survey cluster prior to the start of fieldwork. The maps identified boundaries of each cluster, landmarks, and buildings to allow data collection teams to locate selected households. The catchment area population of the APEs ranged from a population of 500 to 2000, located 8–25km from the nearest health facility (a small number were beyond 25km). Any APE catchment area exceeding 300 households was broken down into smaller units of 150–200 households and the unit containing the APE health post was selected for the household listing. Within each selected cluster, all households were listed and households with children 2–59 months were identified by asking the household head whether any children in that age range were resident; from those listings, 20 households with at least one child aged 2–59 months were selected using systematic random sampling. Within each selected household, one mother or caregiver was randomly selected to be interviewed regarding recent child illness and care–seeking behaviors.

### Data collection and management

The questionnaire was modeled after the UNICEF Multiple Indicator Cluster Survey (MICS) questionnaire (version 4) and included three modules: household, caregiver, and children under–five [17]. The questionnaire was translated into Portuguese and back–translated independently into English to check the accuracy of the Portuguese translation. Data collectors were provided with pictures of common medicines to serve as an aid for respondents to answer questions on medicines received during illness episodes. Trained study staff administered the questionnaire to the caregiver in each household selected for inclusion in the survey. The questionnaire was administered in Portuguese, or orally translated by the interviewer to Macua or Coti, the local languages of survey areas in Nampula province. Interviewers were taught how to correctly translate the survey questions to Macua or Coti during the training.

Data collection was led by the National Institute of Statistics (Instituto Nacional de Estatisticas (INE) in Portuguese) with technical support from SC. Data collectors with prior experience of conducting national household surveys were recruited and trained for six days, including one day of field practice. Data collection was completed in October and November 2012 by four teams of five members each, including a team leader trained to supervise the data collectors, review completed questionnaires, and perform the household sampling. Two field coordinators from INE and two from SC monitored data collection to ensure quality control of data. Data were entered into CSPro by a team of four data entry clerks and double data entry was used to ensure quality; any data discrepancies were reconciled prior to analysis.

### Data analysis

The data were analyzed in Stata IC 11.1 (STATA Corp LLP, College Station, Texas, USA). Data analysis involved calculating frequencies and cross tabulations of care seeking, coverage and consistency of care indicators (**Box 1**). We generated 95% confidence intervals (CI) for each, adjusted for clustering.

Box 1 **Indicators of care–seeking, coverage, and consistency of care**

**Care–seeking and treatment coverage: intervention vs comparison areas**

- Care–seeking: Proportion of children 6–59 months with fever in the last two weeks taken to an appropriate provider (formal public or private providers).
- Treatment coverage: Proportion of children 6–59 months with fever in the last two weeks who received AL.
- Timely and appropriate treatment coverage: Proportion of children 6–59 months with fever in the last two weeks who received AL within 24 hours of onset of symptoms.

**Consistency of reported care with standard case management protocols: APEs vs first–line health facilities as first place of care**

***Appropriate assessment:***

- Respiratory rate assessment for cough or difficult breathing: proportion of children aged 2–59 months with cough and fast/difficult breathing whose respiratory rate was assessed.
- RDT for fever: Proportion of children aged 6–59 months presenting with fever who were administered an RDT

***Communication of results:***

- RDT result communication: Proportion of children 6–59 months presenting with fever who were administered an RDT and whose caregiver were told the results of the test.

***Appropriate treatment of fever:***

- Correct medication: proportion of children 6–59 months with fever who received AL.
- Rationale use of antibiotics: proportion of children 6–59 months with fever only who did not receive antibiotics.
- Timely treatment: proportion of children 6–59 months with fever receiving AL who initiated treatment within 24 hours of onset of symptoms.
- Correct duration: proportion of children 6–59 months with fever receiving AL who took AL for the recommended for 3 days.
- Overall appropriate treatment: proportion of children 6–59 months with fever who initiated AL treatment within 24 hours and took medication for three days.
  ***Provision of first dose and follow-up of children with fever treated by an APE:***
- First dose: proportion of children 6–59 months who received AL from APE who took first dose in presence of APE.
- Follow–up: proportion of children 6–59 months who received AL from APE who returned for follow–up.

#### Indicators of care–seeking and treatment coverage

We applied a standard set of metrics captured in household surveys to compare levels of care–seeking and treatment coverage in intervention and comparison areas (**Box 1**). All results focus on assessment and treatment of children aged 6–59 months as indicated by the national iCCM protocol with the exception of assessment of cough/difficult breathing, which is assessed for children aged 2–59 months. We report results for care–seeking and treatment coverage for fever, which was the focus of project, particularly in terms of supply chain support. We compare areas with iCCM to areas without iCCM.

#### Indicators for consistency of reported care with case management protocol

Using report from mothers or caregivers of children under–five during the household interviews, we assessed indicators of consistency of care defined based on standard case management protocols for children presenting with fever or cough/difficulty breathing in the last two weeks who were managed by APEs and by first level facilities (**Box 1**). Additionally, for children presenting with fever, we measured communication of RDT results and treatment to the caregiver. Under the iCCM protocol, APEs were trained to administer the first dose of treatment and to encourage caregivers to return for follow–up when children have a positive RDT. We assessed consistency with these aspects of the protocol for children treated for fever by an APE with the indicators measuring first dose and follow–up.

This is a comparison based on first source of care. Our analysis was restricted to all children with reported fever and/or cough/difficulty breathing in the two weeks prior to the household survey for which the caregiver sought care first from either an APE or first–line government health facility. Sick children (n = 13) taken first to private clinics, pharmacies, drug shops, informal care providers, or public sector hospitals were excluded from the analysis to allow direct comparison of first level facilities with APEs and sick children who were taken for subsequent care after visiting an APE of first–line government facility (n = 25) were excluded as it would not be possible to differentiate clearly what care was provided by what provider.

### Ethical considerations

Ethical clearance was obtained for the survey from the Ministry of Health Bioethics Committee in Mozambique. Informed oral consent was obtained from every respondent and documented by interviewers on the survey tools.

## RESULTS

Among the 1200 households surveyed with at least one child aged 2–59 months, information was collected for 1531 (753 intervention and 778 comparison) among 1534 eligible children. Characteristics of households are presented in **Table 1**. The intervention and comparison districts had similar household characteristics (**Table 1**). The majority of households in both study areas had a male head of household and fewer than six members. The intervention and comparison areas did differ significantly in caregiver marital and education status, with the caregiver being more likely to be married, and educated in the intervention area.

**Table 1:** Characteristics of intervention and comparison areas within Nampula province, Mozambique

Household characteristics	Intervention area	Comparison area	*P*–value
N = 600	N = 600	
**Male head of household**	83.3%	85.7%	0.3549
**Household size:**			0.5136
1–5 members	63.0%	61.2%	
6 or more members	37.0%	38.8%	
**Marital status of caregiver:**			0.0112
Married	88.2%	80.9%	
Single/separated/widowed	11.2%	19.1%	
**Education of caregiver:**			0.0257
None	50.3%	59.3%	
Primary	45.0%	34.8%	
Middle or higher	4.7%	5.8%	

### Contribution of iCCM to care–seeking and treatment coverage

**Figure 2:**
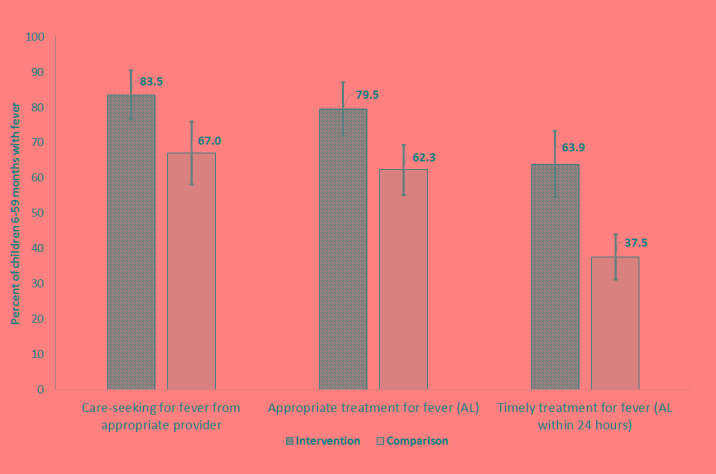
Care–seeking and treatment for fever in intervention and comparison areas, Nampula province, Mozambique

A total of 773 children 6–59 months (346 intervention and 427 comparison) were reported to have fever in the two weeks before the survey. Levels of appropriate care–seeking from formal public or private providers for fever were significantly higher in areas with iCCM compared to those without iCCM (83.5%; 95% CI 76.7–90.4 compared to 67.0%; 95% CI 58.7–75.2; *P* = 0.003) (**Figure 2**). In areas with iCCM, treatment with appropriate antimalarial (AL) was significantly higher than comparison areas (79.5%; 95% CI 72.0–87.0 compared to 62.3%; 95% CI 55.2–69.4; *P* = 0.007). Among those seeking care for fever from a formal public or private source, 87.9% (95% CI 82.6–93.2) received AL in intervention areas compared to 74.1% (95%CI 66.0–82.2) in the comparison areas. The largest differences between intervention and comparison areas were seen in the timeliness of treatment initiation. In iCCM areas where APEs were providing care in the community, 63.9% (95% CI 54.4–73.3) of children with fever initiated AL treatment within 24 hours of symptom onset whereas in comparison areas 37.5% (95% CI 31.1–43.9) of children with fever initiated AL treatment within 24 hours (*P* = 0.000). APEs were the predominant source of care and treatment in the intervention areas; 87.1% (95% CI 80.8–93.4) of children 6–59 months with fever who sought care were taken first to an APE, who accounted for 86.2% (95% CI 79.7–92.7) of all first–line antimalarial treatments. Public health facilities were the leading source of care in comparison areas, providing care to 86.1% (95% CI 79.0–93.3) of children 6–59 months with fever who were taken for care outside the home.

### Consistency of care from APEs and first level facilities with standard case management protocols

We assessed the consistency of reported care with standard IMCI/iCCM protocols among children presenting with fever or cough/difficulty breathing managed by an APE and by first level health facilities (**Table 2**). About two–thirds (68.1%; 95% CI 57.2–79.0%) of children presenting with fever to an APE (N = 248) were tested for malaria with an RDT and 60.0% (95% CI 45.4–74.6%) of children with cough/difficult breathing had their respiratory rate assessed by an APE. In contrast, less than half of children 6–59 months with fever (41.4%; 95% CI 33.7–49.2%) receiving care from a first level health facility (N = 292) were administered an RDT and only 19.4% (95% CI 8.4–30.5%) of children with cough/difficulty breathing were assessed for respiratory rate. A significantly higher percentage of caregivers were counseled on the RDT results by the APE (99.4%; 95% CI 98.2–100%) compared to first level facility staff (78.4%; 95% CI 70.7–86.2%). Children receiving AL from APEs were significantly more likely to initiate antimalarial treatment within 24 hours (84.1%; 95% CI 77.6–90.7%) compared to those seeking care first and receiving AL from a health facility (56.9%; 95% CI 49.9–64.0%). Reported adherence to the recommended treatment duration of three days was also significantly higher for those receiving treatment from APEs; 77.4% (95% CI 68.3–86.4%) of children receiving AL from an APE reported taking AL for 3 days compared with 54.6% (95% CI 44.3–65.0%) of those receiving AL from a first level facility. Overall, 61.3% (95% CI 51.5–71.0%) of children with fever receiving care from APEs received the correct drug within the recommended timeframe and for the correct duration compared to 26.0% (95% CI 18.2–33.9%) of those receiving care from first level health facilities. Inappropriate treatment with antibiotics for fever only cases was low (<10%) for children treated either by APEs or health facilities (**Table 2**).

**Table 2:** Consistency with standard case management protocols by first source of care, Nampula province, Mozambique

Indicators	APE				First level health facility			*P*–value
**N**	**%**	**95% CI**		**N**	**%**	**95% CI**	
**Assessment:**								
Respiratory rate assessment: Proportion of children 2–59 months with cough and fast/difficult breathing whose respiratory rate was assessed with timer	70	60.0	45.4–74.6		144	19.4	8.4–30.5	0.0001
Fever tested with RDT: Proportion of children 6–59 months with fever who were given an RDT	248	68.1	57.2–79.0		292	41.4	33.7–49.2	0.0001
**Communication of results:**								
RDT result communication: Proportion of children 6–59 months who received an RDT and whose caregiver was told the results of the test	169	99.4	98.2–100.7		116	78.4	70.7–86.2	<0.0001
**Treatment:**								
Correct medication: Proportion of children 6–59 months with fever who received AL	248	89.1	83.4–94.8		292	74.0	66.3–81.7	0.0100
Rational use of antibiotics: Proportion of children 6–59 months with fever only who did not receive antibiotics	66	97.0	92.4–101.5		156	92.9	87.0–98.9	0.2911
Timely treatment: Proportion of children 6–59 months with fever receiving AL who initiated treatment within 24 hours of symptom onset	221	84.1	77.6–90.7		216	56.9	49.9–64.0	<0.0001
Correct duration: Proportion of children 6–59 mo with fever receiving AL who took for 3 days	221	77.4	68.3–86.4		216	54.6	44.3–65.0	0.0013
Overall appropriate treatment: Proportion of children 6–59 months with fever who initiated AL treatment within 24 hours and took for 3 days	248	61.3	51.5–71.0		292	26.0	18.2–33.9	<0.0001
**First dose and follow–up:**								
First dose: Proportion of children 6–59 months who received AL from APE who took first dose in presence of APE	221	64.3	53.9–74.6			–	–	NA
Follow–up: Proportion of children 6–59 months who received AL from APE who returned for follow–up	221	70.1	62.9–77.4			–	–	NA

APE – Agentes Polivalentes Elementares, CI – confidence interval, AL – artemether–lumefantrine, RDT – rapid diagnostic test, NA – not applicable

We also looked at the results for the subset of children 6–59 months with RDT+ results. Of caregivers who received the RDT results, reported malaria positivity levels were high (96.4% of those treated by APEs; 95% CI 93.8–99.1 and 93.5% of those treated by health facilities; 95% CI 88.3–98.6). Due to the higher rates of RDT testing and disclosure of results among children managed by APEs, 65.3% of children with fever cared for by APEs in the sample (162/248) were reported RDT+ compared to 29.5% of children with fever cared for by first level health facilities (86/292). Nearly all RDT+ cases received AL (97.5% of those treated by APEs and 94.2% of those treated by health facilities), but RDT+ children who received AL from APEs were significantly more likely to receive AL within 24 hours (88.0% compared to 59.3%; *P* = 0.000) and to take AL for the recommended 3 days (85.4% compared to 63.0%; *P* = 0.005). Overall, 74.1% (95% CI 64.6–83.6) of RDT+ cases managed by APEs received the correct drug within 24 hours and took for 3 days compared with 36.0% (95% CI 21.0–51.0) of RDT+ cases managed by health facilities. The number of RDT– cases was too small (6 for APEs and 6 for health facilities) to analyze.

Under Mozambique’s iCCM protocol, APEs were trained to administer the first dose of treatment to the child and to counsel caregivers to return for follow–up. Of children receiving AL from an APE, caregivers reported that 64.3% (95% CI 53.9–74.6%) took the first dose in the presence of the APE and 70.1% (95% CI 62.9–77.4%) returned to the APE for follow–up. Data were not available for first dose or follow–up for children with fever managed by health facilities.

### Discussion

Our results demonstrate that trained and well–supported APEs implementing iCCM can contribute substantially to timely care and treatment of childhood illnesses in rural Mozambique. A cross–sectional household survey conducted after approximately two years of iCCM implementation showed higher levels of appropriate care–seeking in areas with iCCM services compared to similar areas without iCCM services. Critically, the provision of iCCM by community–based APEs contributed to timely treatment for fever, likely due in large part to bringing curative care closer to the home and reducing delays in seeking appropriate care. Timely treatment is especially important for children with symptoms of malaria and pneumonia, where treatment within 24 hours of symptom onset is linked to improved outcomes [18,19]. Children living in comparison areas who sought care for fever from formal public or private sources were less likely to receive treatment with AL. This could be due to medicine shortages given that the peripheral public facilities providing the majority of care in comparsion areas were reliant on the relatively weak MOH supply chain where stock–outs of essential health supplies were common during the study period [20]. Our results showed that in areas with access to iCCM, utilization of APEs as first source of care was high with APEs providing care for greater than 80% of children with fever. With more cases treated at the community level, iCCM also contributes to reduced work burden at health facilities, which are often overstretched [14,21].

Our findings provide evidence that well–supported APEs can deliver care consistent with country iCCM protocols. In study areas, the APEs provided care aligned with national guidelines and performed as well as or better than facility–based providers for measures of appropriate assessment, counselling and treatment. Children with fever taken first to APEs were significantly more likely to receive an RDT, to be counselled on the RDT results, and to receive treatment with AL than children taken to first–level facilities. Children receiving care from APEs were also more likely to have their respiratory rate assessed and inappropriate treatment with antibiotics for fever only was low, helping to allay concerns regarding the ability of CHWs to manage suspected pneumonia [22]. However, many caregivers visiting APEs for cough did not report their child’s repiratory rate was assessed, indicating that the importance of conducting this assessment step should be reinforced and monitored. Our findings showed that adherence to the recommended malaria treatment duration was significantly better among those treated by APEs than by health facilities; APEs, who are selected by their communities, may be more effective than facility staff in conveying messages to caregivers regarding treatment due to a smaller case load and a closer connection to the community itself. Although based on reports from mothers or caretakers of children under–five, these findings compare well with other recent studies of CHW quality of care in Malawi and Ethiopia, which directly observed CHWs treating sick children and found that CHWs performed well compared to a gold standard clinical re–examiner in assessment, classification, and treatment of iCCM conditions [8,9].

These results were achieved within a well–supported APE program, which is critical for interpreting the findings in the context of the future of iCCM programs implemented by MOH with minimal external support. The iCCM program in Nampula was implemented with support from Save the Children, which provided technical staff and transport to conduct monthly onsite supervision of APEs. In addition to supervision, the APEs in the SC program areas were well supported with a reliable supply of materials and medications (and not reliant on MOH supply chain). Program data collected through interviews, observations and record review for the 30 APEs in the selected intervention clusters alongside the household survey indicated that the majority of APEs had the necessary supplies and medicines for iCCM provision: 90% had a functional timer; 87% had RDTs in stock; 80% had AL in stock; and 80% had first–line antibiotics available [23]. Supervision was frequent, with 80% of APEs reporting that they had been supervised in the previous month [23]. This illustrates that the positive findings reported here can only be sustained at scale with a well functioning national supply chain and other support systems in Mozambique. In 2013, SC intensive supervision and supply chain support for iCCM was phased out and management of the iCCM supply chain was handed over to the MOH, through which APEs are resupplied with a standard “kit” (push system) rather than based on reported consumption. This reduced external supply chain support, coupled with national stock–outs of AL and the use of a “kit” system, has resulted in pervasive stock–outs among APEs of critical iCCM supplies.

Our findings highlight the need for greater attention to the management of childhood illness at first level facilities and increased investment in broader health systems strengthening beyond short–term, mostly vertical supports [24]. Poor adherence to IMCI protocols at first level facilities has been reported in similar settings in which case management was directly observed [25,26]. In a study conducted in Ghana, just 4% of children presenting to health centers and district hospitals with cough or difficult breathing had their respiratory rate assessed and in only 1% of all cases were all 11 expected tasks performed [25]. Similar results were found in a study of children in Tanzaniawith acute respiratory infections in which just 5% of children with cough or difficult breathing had their respiratory rate counted at two district hospitals [26]. It was not possible to determine from our study whether lack of training, lack of supplies, poor motivation or a combination of these factors were responsible for the performance gaps at first level health facilities. Health facilities were supplied with AL through the provincial and district medical stores based on consumption reports; facility assessments conducted in Sofala province around the same time period revealed that stock–outs of AL were frequent, particularly among smaller and more rural faciltiies [20]. Furthermore, while RDTs were introduced in 2007 to peripheral health facilities in Mozambique, some staff may not have been well–trained or confident in their use and RDT shortages were reportedly common, due to poor consumption tracking and weak design of the supply chain system that failed to address seasonality of malaria [27]. Lack of supplies is unlikely to be the primary reason for the low levels of respiratory rate assessment, as providers could use a variety of devices for this purpose (cell phones, watches), one of which is typically on hand. The level of reported antibiotic prescription for fever only cases managed by facility providers was slightly higher than among APEs, but still relatively low and may have been underestimated; a 2011 review of patient records for febrile children in Zambia found that prescription of antibiotics for fever cases was reduced when RDT results were availabile, suggesting that greater availability of RDTs could help reduce irrational use of antibiotics [28]. The lack of adherence to case management protocols observed among facility–based providers is cause for concern given that these providers are often tasked with supervision of CHWs implementing iCCM. This finding reinforces the need to strengthen knowledge and skills of facility–based staff through routine supervision and on–the–job training, strengthen the national supply chain to ensure adequate availability of essential supplies and equipment, and address other performance–related factors [24–28].

This study is one of the first to our knowledge to use household surveys to assess aspects of consistency with iCCM/IMCI protocols. Specifically, the indicators on counting of respiratory rate, communication of RDT results, provision of first dose, and follow–up visits have not previously been measured in standard household surveys. Although these indicators have not been validated, they ask caregivers to recall noteworthy events during the examination and/or treatment course for a relatively short recall period of two weeks. A register review of activity documentation by APEs from the 30 intervention clusters found similar levels of recorded respiratory rate counting and RDT administration to that reported by caregivers during the household survey; 87% of APEs had RDT test results recorded for the five most recent cases of fever and 63% had respiratory rates documented for the five most recent cough cases [23]. We propose that these indicators be included in household surveys assessing case management for child illness and further validated, as they adequately capture provider activities and enhance the knowledge of patient–provider interaction.

Several limitations should be recognized when considering our findings. The study design was constrained by limited funding and time for evaluation and we did not have a baseline against which to measure change over time. To obtain a sufficient sample size, we only sampled households in which a child aged 2–59 months was identified during household listing. Although care was taken to identify all such households during the household listing stage, errors in age measurement or absence of any respondent in these households may have excluded some eligible households from the sample. Additionally, our sample relied on the comparison of the intervention districts to one comparison district. This sampling strategy was necessary given the rapid national roll–out of iCCM in Mozambique, but can make the results challenging to interpret as one cannot quantify or account for the potential influence of local contextual differences [29]. Although relatively small, the differences in education level between the intervention and comparison area respondents may have affected reported care–seeking practices and treatment. Consistency with case management protocols was determined based on caregiver recall, which is imperfect, and cannot be used to determine quality of care comparable to other methods such as direct observation/re–examination or record reviews [30]. Although assessing of respiratory rate is noteworthy in that it requires the health worker to use a timing device and lift the child’s shirt, caregivers would not be able to tell whether the respiratory rate was calculated correctly and whether the provider made the correct classification. Similarly, for RDT use, it was not possible to determine based on caregiver report whether the RDT was administered and interpreted correctly. In addition, for fever we were unable to fully account for the differential in RDT use between APEs and the health facilities, which could also result in differences in the treatment indicators. However, sub–analysis restricted to RDT+ cases revealed similar findings as that for all fever cases. Furthermore, we were unable to assess the quality of counseling APEs provided to the caregivers. However, high reported adherence to medication duration and high rates of return for follow–up suggest that key messages were conveyed appropriately. We excluded the small number of sick children taken first to private clinics and pharmacies, and as such cannot comment on the consistency of care provided through these other sources of care, which can be important sources particularly in more urban areas.

## CONCLUSION

This study demonstrates that well–trained and supported APEs providing iCCM significantly contributed to improved timely and appropriate treatment of fever in rural Mozambique. Demand for iCCM was strong as evidenced by high levels of utilization of APEs as first source of care in program areas. In addition, APEs provided care that was consistent with iCCM protocols and performed significantly better than first level facilities on most measures of adherence to case management protocols. These findings reinforce the need for comprehensive efforts to strengthen the health system in Mozambique to enable reliable support for quality of case management of childhood illness at both health facility and community levels.
